# Functional Cohesion of Gene Sets Determined by Latent Semantic Indexing of PubMed Abstracts

**DOI:** 10.1371/journal.pone.0018851

**Published:** 2011-04-14

**Authors:** Lijing Xu, Nicholas Furlotte, Yunyue Lin, Kevin Heinrich, Michael W. Berry, Ebenezer O. George, Ramin Homayouni

**Affiliations:** 1 Bioinformatics Program, University of Memphis, Memphis, Tennessee, United States of America; 2 Department of Mathematical Sciences, University of Memphis, Memphis, Tennessee, United States of America; 3 Department of Computer Science, University of Memphis, Memphis, Tennessee, United States of America; 4 Computable Genomix, Memphis, Tennessee, United States of America; 5 Department of Electrical and Computer Engineering, University of Tennessee, Knoxville, Tennessee, United States of America; 6 Department of Biological Sciences, University of Memphis, Memphis, Tennessee, United States of America; Cairo University, Egypt

## Abstract

High-throughput genomic technologies enable researchers to identify genes that are co-regulated with respect to specific experimental conditions. Numerous statistical approaches have been developed to identify differentially expressed genes. Because each approach can produce distinct gene sets, it is difficult for biologists to determine which statistical approach yields biologically relevant gene sets and is appropriate for their study. To address this issue, we implemented Latent Semantic Indexing (LSI) to determine the functional coherence of gene sets. An LSI model was built using over 1 million Medline abstracts for over 20,000 mouse and human genes annotated in Entrez Gene. The gene-to-gene LSI-derived similarities were used to calculate a literature cohesion p-value (LPv) for a given gene set using a Fisher's exact test. We tested this method against genes in more than 6,000 functional pathways annotated in Gene Ontology (GO) and found that approximately 75% of gene sets in GO biological process category and 90% of the gene sets in GO molecular function and cellular component categories were functionally cohesive (LPv<0.05). These results indicate that the LPv methodology is both robust and accurate. Application of this method to previously published microarray datasets demonstrated that LPv can be helpful in selecting the appropriate feature extraction methods. To enable real-time calculation of LPv for mouse or human gene sets, we developed a web tool called Gene-set Cohesion Analysis Tool (GCAT). GCAT can complement other gene set enrichment approaches by determining the overall functional cohesion of data sets, taking into account both explicit and implicit gene interactions reported in the biomedical literature.

**Availability:**

GCAT is freely available at http://binf1.memphis.edu/gcat

## Introduction

Microarray technologies are routinely used to examine gene expression profiles under different experimental conditions. However, statistical analysis of microarray experiments still remains challenging, due in part to sensitivity (low signal to noise) of the method as well as technical, biological and multiple testing confounds. A large amount of effort has focused on developing mathematical models to normalize and identify differentially expressed genes [1]. Simulation studies can be used to measure the performance of different statistical methods [2]. However, these studies have limitations on sample sizes and uncertainty in degree of conformability between stimulated datasets and real microarray data. Jeffery and coworkers [3] compared gene sets generated by 10 different feature selection methods, for example, significance of microarrays (SAM), analysis of variance (ANOVA), empirical Bayes t-statistics, and found that there was a big discrepancy in gene sets produced by different algorithms. Most importantly, these methods did not include any functional (biological) information to evaluate differentially expressed gene sets.

To incorporate biological information into algorithms for identification of meaningful gene sets, most of the existing methods utilize functional category enrichment analysis based on Gene Ontology (GO) [4]–[7]. GO contains a structured, precisely defined, controlled vocabulary for describing the role of genes and gene products in any organism [8]. Although the standard enrichment methods are useful to interpret the common function in a group of genes/proteins, these methods have certain drawbacks. First, each GO term is treated independently by these methods; hence, associations among multiple GO terms are ignored. Second, it is hard to evaluate the overall significance of functional cohesion within a gene/protein group when multiple GO terms are enriched [9].

In recent years, a growing number of studies have focused on estimating the literature-based functional coherence of gene groups. In 2002, Raychaudhuri [10] developed the neighbor divergence per gene (NDPG) method, which uses natural language processing (NLP) to extract gene information from the biomedical literature. NPDG estimates functional cohesion by comparing the difference between the empirical and theoretical distributions of coherence scores using Kullback-Leibler divergence [11]. This method was evaluated using 2,796 GO gene sets from yeast, mouse, fly and worm. High sensitivity was obtained with each of these organisms except worm. As pointed out by Zheng and Lu (2007a), statistical significance calculated by NPDG may be problematic since the divergence of Kullback-Leibler is not normally distributed. They proposed that by association of literature-derived protein information with biological concepts in GO, the degree of functional similarity among protein groups can be evaluated more accurately [12]. By estimating the mean and variance of the coherence score from random groups of proteins, they calculated the p-value of the observed coherence score based on the asymptotically normal distribution function. Later, Chagoyen et al. used the pair-wise similarities of functional annotations from GO to calculate the coherence score of a protein set. The significance assessment of the coherence score was performed by measuring the functional relatedness of a given protein set compared with another set drawn from a reference set [13]. The latter methods have limitations imposed by GO, which has a limited range of functional categories and is human curated.

Previously, we developed a method which utilized Latent Semantic Indexing (LSI), a variant of the vector space model of information retrieval, to determine the conceptual relationships between genes from information in MEDLINE titles and abstracts [14]. This method was shown to be robust in identifying both explicit and implicit gene relationships. In the present study, we developed a new method to calculate the functional coherence of gene sets using LSI-derived similarities among the genes. Using a Fisher's exact test, we calculate the significance of functional connectivity derived from literature in a given gene set compared to that expected by chance. We conducted a large-scale evaluation of our method against the functional gene groups in GO and demonstrated the application of the method to microarray expression data. Importantly, we developed a unique web tool that can calculate the literature cohesion of gene sets in real time, which will enable researchers to compare feature selection methodologies and to hone down large gene sets into more manageable sizes for further investigation.

## Methods

### Gene-document collection

Each gene document was constructed by concatenation of all titles and abstracts of the Medline citations cross-referenced in the mouse, rat and human Entrez Gene entries as of 2007. Gene information was downloaded from the National Center for Biotechnology and Information site (ftp://ftp.ncbi.nlm.nih.gov/gene/DATA). PubMed IDs (PMIDs) corresponding to homologous genes were combined. Orthology data were downloaded from Mouse Genome Informatics site. To reduce false positives, PMIDs referring to more than 10 genes were removed as these citations usually described genomic experiments and contained no functional information. After filtering, there were 17,451 human and 21,903 mouse genes in our gene document collection (Table 1). A few genes had many abstracts, with the maximum number reaching 2,923. The average number of abstracts for each gene was 23.6 for human and 19.5 for mouse.

**Table 1:** Summary of mouse and human gene document collections.

	# of genes	Abstracts range	Median # of abstracts	Mean # of abstracts	# of genes with 1 abstract
Mouse	21903	1∼2923	3	19.5	6436
Human	17451	1∼2923	5	23.6	4343

### Generation of the similarity matrix

The method to calculate gene-gene similarity scores using LSI was described by our group previously [14], [15] and is summarized briefly here. A term-by-gene matrix was created for mouse and human genes where the entries of the matrix were the weighted frequencies of terms across the entire collection. A reduced rank term-by-gene matrix was generated by computing the singular value decomposition (SVD) as described previously [15]. Genes were then represented as vectors in the reduced rank matrix and the similarity between genes was calculated by the cosine of the vector angles. The higher the cosine score, the higher the association between the two gene vectors. A cosine score of 1.0 means that the two genes have exactly the same abstracts in our collection. This is very rare, occurring in ∼0.003% of all gene pairs in the collection. For this study, 300 factors were used to calculate the gene-by-gene similarity scores because it was shown to be an optimal number of dimensions for large document collections [16]. The similarity matrices on the GCAT website will be updated on an annual basis.

### Gene Ontology information

The Gene Ontology data file (version 5.7.2) was downloaded from http://www.geneontology.org/GO.downloads.ontology.shtml. The gene-to-GO category association was based on the annotation provided by Ensembl Version 42 (http://www.ensembl.org). We focused on the GO categories that contained 5 to 1000 genes, which resulted in a total of 6681 GO categories across the human and mouse collections.

### Gene-set cohesion analysis tool

The GCAT software was implemented using a Dell X-series server with quad-core 1.6 GHz Pentium Xeon processors and 8 GB of RAM. Python, MySQL, R and rpy (http://rpy.sourceforge.net/) were utilized to create the web front end and back end. Cytoscape (http://www.cytoscape.org/) was used to create and visualize the gene network graphs. The Scriptaculous (http://script.aculo.us/) and prototype.js javascript libraries were used to create limited visual effects within the web browser. The data for individual genes was obtained from the NCBI ftp site.

## Results

### Overview of LPv algorithm

**Figure 1:**
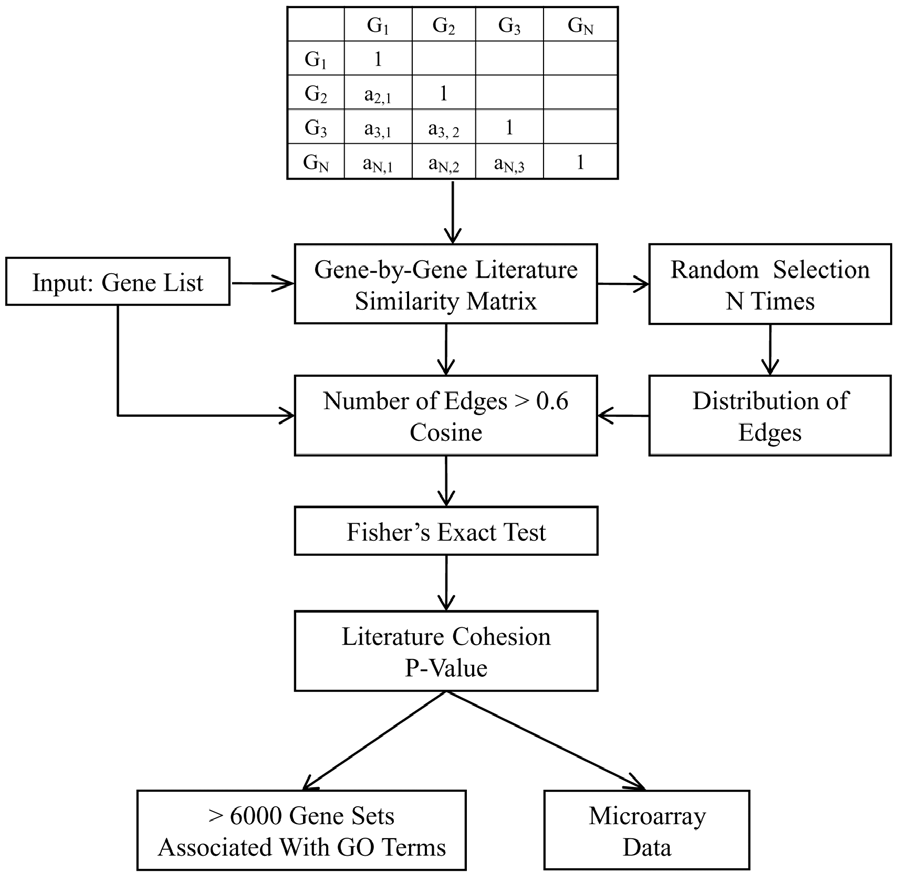
Work flow for Gene-set Cohesion Analysis. A gene-by-gene similarity matrix was constructed for >20,000 mammalian genes using LSI on Medline abstracts cross-referenced in Entrez Gene (2007). The cosine similarity threshold was calculated empirically from 1000 randomly selected gene sets so that only the top 5th percentile of edges (PE), i.e., gene relationships, were considered for the analysis. Fisher's exact test was performed to determine if the number of gene relationships above the threshold in a given dataset is significantly different from that which is expected by chance. This method was evaluated by calculating the literature p-value for >6000 gene sets associated with GO terms and applied to analysis of a microarray dataset.

**Figure 2:**
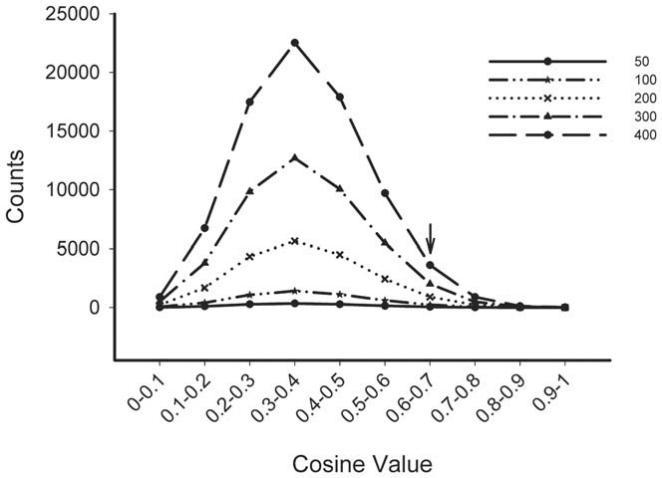
Distribution of cosine similarity scores for randomly selected gene sets ranging from 50 to 400 genes. The cosine value 0.6 (arrow) represents approximately 5% of the total number of cosine scores in the collection.

The workflow for the literature-derived p-value (LPv) procedure is shown in Figure 1. The method aims to determine the significance of the functional cohesion for a given gene set compared to the cohesion of a randomly selected gene set. For testing the significance of cohesion, we compared the observed number of gene relationships above a cosine threshold of 0.6 in the LSI model. This cut-off threshold was chosen based on examination of 1,000 random gene sets (containing between 50–400 genes each) from the gene-by-gene LSI similarity matrix constructed in 2007. The distributions of the similarity scores, which ranged from 0 to 1 for the various sampled gene sets, are shown in Figure 2. We found that approximately 5% (ranging from 5% to 5.8%) of the similarity scores were above a cosine value of 0.6. Therefore, gene sets which have many cosine scores above 0.6 would be considered functionally cohesive. The significance of the functional cohesion was measured by a Fisher's exact test.

### Evaluation of LPv accuracy

The GO database contains greater than 22,000 functional classifications (GO terms) in three major categories: biological process, cellular component and molecular function. Genes are assigned to GO terms by human experts based on published experimental results. We assumed that genes assigned to each GO term share similar functions and treated them as gold standard to evaluate the robustness of the LPv methodology. We calculated LPv for 6,681 mouse and human GO terms that contained between 5 and 1,000 genes (Table 2). The average number of genes linked to each GO term for all three broad GO categories ranged from 49 to 66. The proportion of gene sets in each GO category which showed an LPv <0.05 is shown in Table 2. Our method achieved 91.3% and 91.6% accuracy for GO terms in molecular function for mouse and human collections, respectively. In addition, 90.6% (mouse) and 89.9% (human) of the gene sets in cellular component were found to be significantly cohesive. However, the biological process category had the lowest percentage (about 75%) of groups which were found to be cohesive by our method. These results are consistent with those reported by Raychauduri et al. [17], who used NDPG to evaluate the functional coherence of the gene sets in each GO category.

**Table 2:** Performance of LPv methodology on gene sets in GO categories.

Mouse	# of Go terms	Avg # of genes	LPv<0.05
biological process	1891	53.9	75.60%
cellular component	384	66.3	90.60%
molecular function	909	48.7	91.30%
Human			
biological process	2046	58.7	74.40%
cellular component	424	65.8	89.90%
molecular function	1027	49	91.60%

The number of gene sets associated with GO terms along with the average number of genes in each major branch is shown for both human and mouse collections. The proportion of gene sets that contained an LPv<0.05 is indicated.

**Figure 3:**
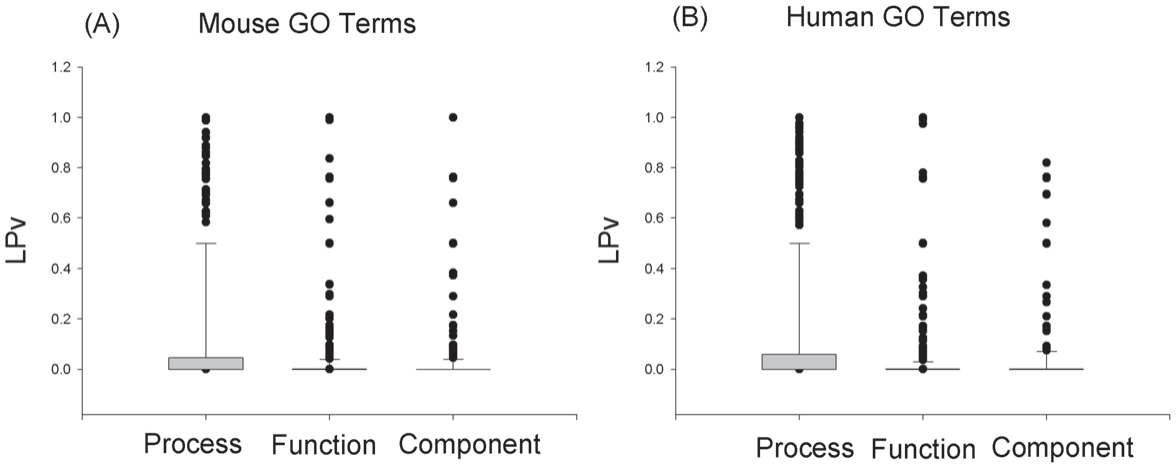
Box plot of LPv distributions for gene sets in the three main mouse and human GO categories.

**Figure 4:**
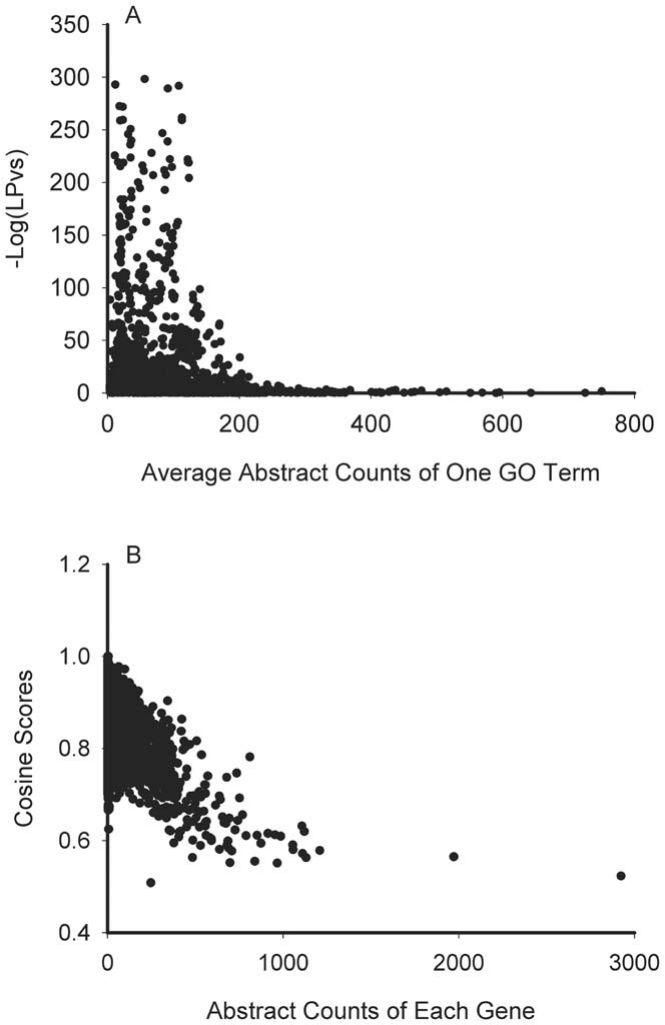
Relationships between abstract counts, LPv, and cosine scores. (A) The average number of abstracts for each gene set in GO was plotted against the −Log(LPv) for the group. (B) The top 10 cosine scores were averaged for each gene and plotted against the abstract count for the gene.

Examination of the LPv distributions of the three main GO categories (Figure 3) revealed that the biological process category had many more outliers than the other two GO categories. One possible explanation for the outlier groups may be that some biological processes contain genes that are associated with many different pathways in the literature, resulting in a low cosine score with other genes. We found that the average number of abstracts for GO terms with LPv>0.05 was 146.8 compared to 80.1 for GO terms with LPv<0.05. Indeed, there is a positive correlation between the LPv and number of abstracts in a gene set (Figure 4A). In general, the smaller the LPv (high −Log (LPv) in the figure), fewer the number of abstracts were associated with the gene set. In addition, we examined the correlation between the number of abstracts and the top 10 cosine values (neighbors) for a given gene (Figure 4B). We found that the higher the number of abstracts associated with a gene, the lower the overall average cosine value of its top 10 gene neighbors.

Table 3 lists the number of abstracts and the average cosine score for the top most well-studied genes in our collection. As expected, these well-studied genes appeared in the biological process category more frequently than in the other two GO categories. For example, Tnf appeared in 280 GO terms in biological process (15% of the total biological process terms), but in only 22 (2.4%) and 11 (2.9%) in molecular function and cellular component, respectively. This could be one of the reasons that we observed fewer biological process terms with significant LPvs (false negatives) than with molecular function and cellular component terms.

**Table 3:** Genes with greater than 1000 abstracts and the average cosine scores for their top 10 gene neighbors.

Gene	# of abstracts	Cosine scores
Trp53	2923	0.5482
Tnf	1971	0.5772
Tgfb1	1208	0.6074
Fas	1130	0.599
Vegfa	1120	0.6478
Apoe	1110	0.594
Ifng	1106	0.6542
Il6	1056	0.6028
Lep	1054	0.6178

### Evaluation of LPv sensitivity

**Figure 5:**
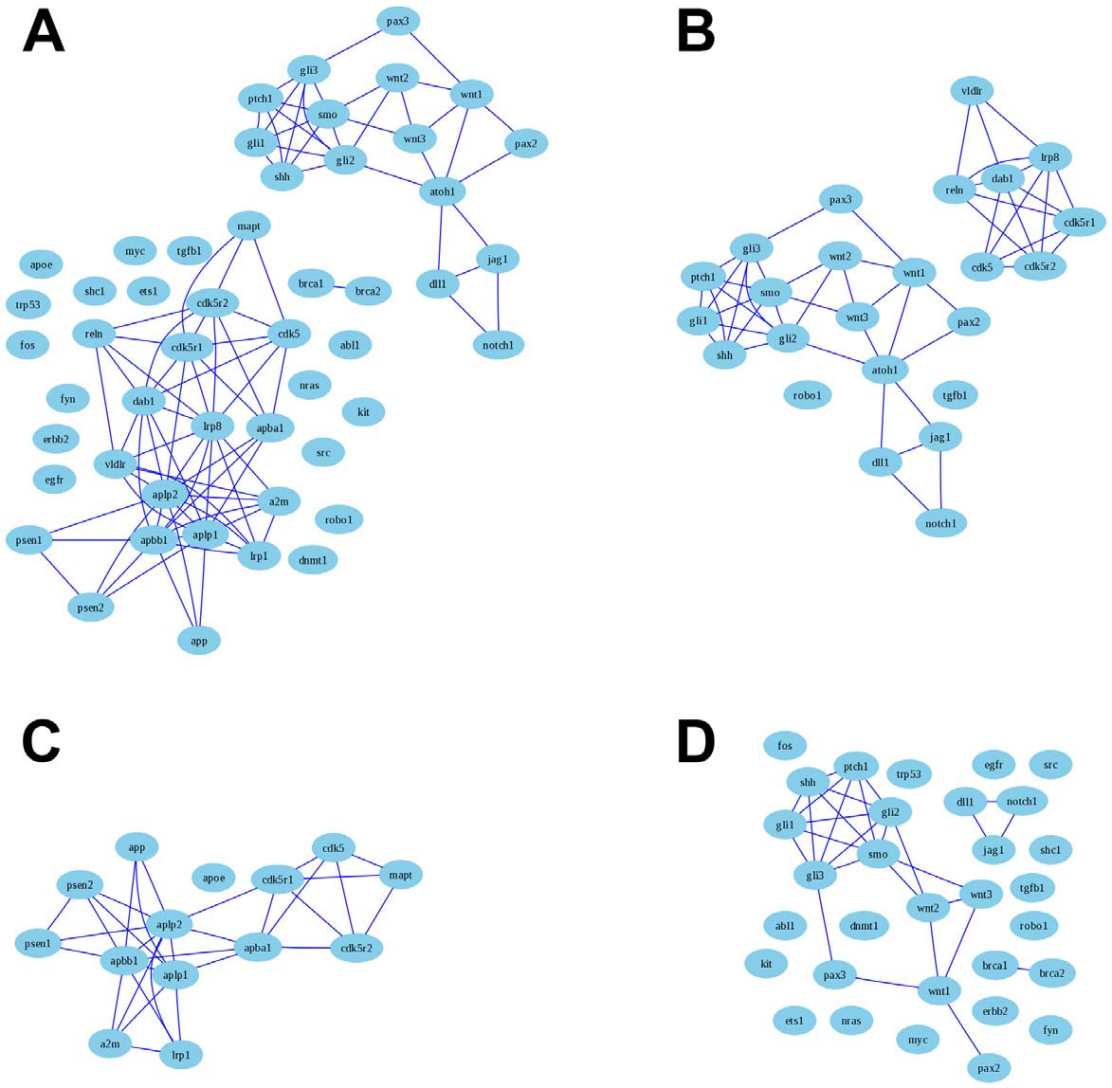
A network graph representation of literature relationships among 50 manually selected genes (A). Graphs are also displayed for functional categories: development (B), Alzheimer's disease (C) and cancer (D) as described previously in [14]. Nodes in the graphs represent individual genes and the edge threshold is a cosine of 0.6, which represents approximately 5% of all edges in the collection.

To investigate the sensitivity of LPv methodology, we examined 50 manually selected genes related to three functional categories: development, Alzheimer disease (AD) and cancer [14]. The LPv for this 50-gene set was 0.00777, suggesting that the genes in this set were functionally cohesive. The network graph for the 50-gene set indicated that there were three sub-clusters containing the genes associated with the three functional categories (Figure 5). Interestingly, the three subsets within the 50-gene collection produced LPv of 9.5×10^−7^, 7.1×10^−8^ and 0.282 for development, AD and cancer, respectively.

The genes in both the development and Alzheimer disease groups were highly connected, whereas the genes in the cancer group were mostly disconnected, causing the LPv of the cancer gene set to be insignificant. The average number of abstracts associated with each group was 229, 243, and 439 for development, AD and Cancer, respectively. These results indicate that the LPv method can tolerate bias created by well-studied genes in even small subsets of genes, since inclusion of Trp53 (and others) in the cancer field still produced a significant LPv for the 50-gene set.

### Gene-set cohesion analysis tool (GCAT)

A major goal of this study was to produce an automated and robust method to evaluate the functional coherence of (or literature support for) gene lists derived from genomic studies. Thus, we developed a web tool called Gene-set Cohesion Analysis Tool (GCAT) to enable calculation of LPv on the fly (http://binf1.memphis.edu/gcat). Users need to simply select the organism (human or mouse), the microarray platform (currently limited to Affymetrix), and input the list of genes by typing or pasting either gene symbols or Entrez Gene IDs. GCAT displays the LPv along with a network graph, where genes are represented as nodes and the edges represent cosine values >0.6. GCAT also provides the LSI similarity scores, which can be downloaded in tabular form, between all genes in the input set. In addition, a gene annotation table is provided, in which the gene IDs are hyperlinked to the Entrez Gene website and the abstract number associated with each gene is hyperlinked to PubMed website. Besides, GCAT displays the common log-entropy terms derived from the gene abstracts which can provide insights into biological functions of the gene set.

### Application of LPv to microarray data

Microarrays allow researchers to measure thousands of transcripts simultaneously and to determine genes whose expression levels are significantly altered by an experimental treatment. However, the false positive rate (FDR) can be very high for a number of reasons, including those associated with multiple hypothesis testing. To reduce false positives, biologist often select differentially expressed genes (DEGs) using a combination of arbitrary criteria such as the lowest expression p-value, highest magnitude of change, and/or a fold change cut off of 2. However, these selection criteria may not necessarily be biologically relevant and could result in loss of true positives. We postulate that by using a literature-based cohesion analysis approach, biologist should be able to have an objective criterion to evaluate various statistical feature selection methods and be able to arrive at a biologically relevant gene set more effectively.

To test the utility of GCAT for microarray analysis, we used a previously published microarray data set of interferon stimulated gene expression in mouse embryonic fibroblasts [18]. We found that among 5,518 genes present on the microarray, 216 genes were differentially expressed after interferon treatment. As expected, after multiple testing corrections [19], no significant DEGs remained. However, manual inspection of the DEGs revealed that at least a third of the genes were well-known interferon stimulated genes, suggesting that multiple testing correction is too stringent in this case. Importantly, 216 interferon stimulated genes had an LPv<6.0×10^−32^, suggesting that they are highly functionally related in the literature (Table 4). Genes that were expressed greater than 2-fold had a smaller LPv (2.4×10^−44^). Moreover, genes that were changed greater than 2-fold and had an expression p-value<0.05 produced the smallest LPv (1.4×10^−63^). In contrast, genes that were changed greater than 2-fold and had an expression p-value>0.05 or genes that were changed less than 2-fold and had an expression p-value<0.05 did not show significant literature cohesion. These results demonstrate the utility of using an objective criteria based on functional information in the literature to fine tune statistical analysis of high-throughput experiments.

**Table 4:** LPv for different gene lists identified by microarray analysis.

Gene selections	# of input genes	# of genes with abstracts	LPv
P<0.05	216	168	6.00E-32
FC>2	164	139	2.42E-44
P<0.05 & FC>2	110	93	1.37E-63
P<0.05 and FC<2	106	75	0.344102
P>0.05 & FC>2	54	46	0.197204

Differentially expressed genes were identified by a combination of fold change or p-value cutoffs after performing a student t-test between an interferon treated and control treated cells.

Next, we explored whether GCAT could be used to compare the results of different feature extraction methods. We applied our method to a previously published acute lymphoblastic leukaemia (ALL) dataset including four different groups [20]. The ALL1 group contained B-cell (n = 95) and T-cell (n = 33) samples. The ALL2 group contained samples from patients with (n = 24) or without (n = 101) multidrug resistance. The ALL3 group contained samples from patients that did (n = 65) or did not relapse (n = 35). Finally, the ALL4 group contained samples from patients with (n = 26) or without (n = 67) the t(9;22) chromosome translocation. Similar to Jeffrey et al., we applied three different methods (Welch's t-statistics, Empirical Bayes statistics and SAM) to the four groups [3]. Table 5 lists the LPvs of the top 100 DEGs generated by each test statistics for each of the four datasets. As expected, we found that different test statistics generated different sets of DEGs (Supplementary Figure S1). For the ALL3 dataset, 63 of the top 100 DEGs identified by each test statistic were the same, indicating that the experiments are likely to be technically and biologically sound. Consistent with this observation, the LPvs for these DEGs were very small (Table 5). In contrast, only 22 genes out of the top 100 DEGs produced by each method were common to either the ALL1 or ALL2 dataset. Interestingly, for each dataset, one test statistic clearly out-performed the others based on the LPv determination. The Bayes method produced biologically cohesive gene sets for ALL1, ALL3, ALL4, whereas Welch's t-test produced the most cohesive gene set for the ALL2 samples. These results demonstrate that by ranking the LPvs, feature selection methods can be evaluated objectively.

**Table 5:** LPvs for gene sets identified by different feature selection methods on four different microarray experiments (ALL1, ALL2, ALL3, and ALL4; see text for details).

Methods	ALL1	ALL2	ALL3	ALL4
Welch	1.10E-02	5.25E-08	3.48E-08	9.00E-02
Bayes	5.47E-12	1.00E+00	2.73E-14	3.00E-03
SAM	6.00E-02	1.00E+00	5.32E-07	3.00E-02
# of common genes	22	22	63	53

## Discussion

In a previous study, we developed a novel method to determine the conceptual relationships between genes directly from Medline abstracts using LSI [14]. Unlike the concept-based approach of Schuemie et al which finds protein-protein interactions [21], LSI defines the similarities of word usage patterns in the gene abstract documents. Using our approach, genes/proteins can be functionally grouped using pairwise distances derived from the literature based on both explicit and implicit relationships. The implicit information extracted from biomedical literature has proven to be very useful for knowledge discovery [22], [23], particularly in genomic applications whereby new gene associations are routinely unveiled [24].

In the present study, LSI-derived gene-gene similarities were used to determine a literature cohesion p-value (LPv) for gene sets. This work is significant because it provides a robust method for researchers to objectively evaluate the literature support for any given gene set derived from high-throughput technologies. The method can assist researchers in choosing appropriate feature selection methodologies or prioritizing multiple gene lists generated by different algorithms.

The accuracy and sensitivity of LPv was evaluated using the gene sets associated with GO terms and a human-curated dataset. We found that most of the gene sets had statistically significant literature cohesion, indicating that our method correctly identifies functional coherence of gene sets. Greater than 90% of the gene sets in the molecular function and cellular component categories had significant LPv. In contrast, only 75% of groups in the biological process category were significant. These results are very similar to those obtained by Raychaudhuri et al [17], who used the NDPG method, to assess the coherence of genes associated with GO terms. Taken together, these results suggest that annotation of some GO terms may not be accurate and that using literature cohesion approaches could assist in curation of the GO database. On the other hand, we found that the lower proportion of significant groups in the biological process may be due in part to the fact that many biological processes include very well-studied genes which have multiple functions. Well-studied genes, having many abstracts, are likely to be assigned to multiple functions and pathways. Consequently, we found that well-studied genes generally had low cosine scores with their top ranked neighbors (Figure 4B). Therefore, the presence of well-studied genes in the subset lowers the average cosine value and, consequently, lowers the proportion of genes in the subset which have a cosine value above the threshold of 0.6 (necessary for calculation of LPv). Importantly, we found that Tnf (along with several other well-studied genes) was assigned to more than 15% of the total number of gene sets in biological process examined in this study. Thus, the inclusion of well-studied genes in multiple biological processes could account for the lack of literature cohesion in some of the GO gene groups.

Although the presence of well-studied genes can negatively impact LPv calculation, the method appears to be quite robust. The LPv of a human-curated set of 50 genes, which included well-studied genes such as Apoe, App, and Trp53 was statistically significant (LPv 0.007), whereas a subset of 31 cancer related genes had LPv of 0.282. This result suggests that cancer is generally well-studied and that genes involved in cancer are associated with many pathways and processes. Therefore, some caution is necessary when interpreting LPv for gene sets which contain a disproportionate amount of well-studied genes. To provide some feedback to the users of GCAT, we display the number of abstracts for each gene and provide a hyperlink to PubMed.

A number of other groups have developed methods to determine literature-based functional coherence of gene/protein sets [9], [10], [12], [13], [17]. Zheng and Lu used GO annotation to extract biological topics that were used to construct a protein-topic association matrix [12]. In their study, the functional coherence was evaluated via the closeness of the proteins in the network. Chagoyen et al. (2008) computed the similarity scores between genes/proteins described as vectors of GO terms [13]. Both of these methods rely on GO annotation, which may limit the scope of gene/protein functions. Furthermore, the information in human-curated indices and databases may not be updated as quickly as in the biomedical literature, which is utilized by our method. For instance, GO analysis of the interferon dataset used in our study identified 2 genes in MHC class II and 8 genes in GTPase categories. However, using an LSI approach, we identified at least one MHC II gene (H2-q7) and one GTPase (Arf3) that was not in GO (data not shown).

Microarray experiments are complex and subject to technical and biological variability that hinder inference analysis. Consequently, different statistical procedures must be carefully considered for each microarray experiment. Numerous statistical methods are available for identifying differentially expressed genes [3], but each has a different set of assumptions and result in a different set of DEGs. Relatively few methods are available to help researchers decide if a particular statistical test is appropriate for the microarray experiment or if it produces functionally relevant gene sets [25], [26]. We demonstrated the utility of LPv for evaluating different analytical methods using several published microarray datasets. With respect to the interferon dataset, we found that the gene list corresponding to p-value<0.05 and fold change above 2 exhibited the smallest LPv compared with the other thresholds examined (Table 4). With respect to the ALL datasets, we found that empirical Bayes statistics was the most biologically (functionally) supported method for the ALL1, ALL3 and ALL4 datasets. On the other hand, Welch's t-test was most appropriate for the ALL2 dataset. As an extension of this application, GCAT could easily be used to compare the performance of different clustering algorithms. Generally, it is hard to determine the optimal clustering solutions for microarray experiments. Yona and colleagues proposed using internal and external indices to determine the best clustering algorithm that maximizes the correlation of functional links and minimizes the error rate [27]. However, the statistical significance was not calculated for determining the most appropriate clustering algorithms. Our LPv analysis could be used to estimate the significance of the functional coherence of individual clusters. Potentially, GCAT could be adapted to rapidly prioritize different clustering algorithms by utilizing information from the biomedical literature.

To our knowledge, one other web tool besides GCAT is available for estimation of statistical significance of protein-protein networks, called Studying Networks in the Omic World (SNOW) [28]. The main difference between GCAT and SNOW is that GCAT uses comprehensive literature to find gene/protein relationships whereas SNOW defines the interaction based on five public databases. In addition, SNOW calculates several parameters of the network derived either from the user or existing databases, and the significance of the network is determined via the Kolmogorov-Smirnov test by comparing with the empirical distribution of the network parameters.

Another program, called Gene Set Enrichment Analysis (GSEA), determines enrichment p-values for the biological or functional categories cataloged in various databases [25], [29]–[32]. Subramanian et al. (2005) used GSEA to assess if genes in functional groups occurred together near the top of a gene list obtained from expression data. One drawback of GSEA is that it is sensitive to the size of the gene set. In addition, GSEA does not reflect the overall functional relationships among all genes/proteins in the gene set and does not consider cross-talk between pathways [33]. Therefore, GSEA cannot be used to evaluate whether the experimental design or methodology is appropriate. In contrast, our LPv method can determine the overall functional coherence of a gene set which may span multiple pathways or classifications. The functional groups in GSEA are drawn from different human-curated databases, which can have a number of limitations such as sparsity, knowledge lag-time, and limited vocabularies. Moreover, these databases contain only explicitly linked information, which ignore implied relationships and new discoveries. Utilization of an LSI approach on biomedical literature circumvents many of these limitations. For instance, in the interferon example mentioned above, our LSI approach identified one additional GTPase along with three GTPase regulators that were not in GO (data not shown). Similarly, LSI identified one additional MHC II class protein along with four regulators of MHC proteins that were not in GO (data not shown). Including regulators of GTPases or MHC class proteins has important functional significance and should be included in gene set analysis and functional cohesion determinations.

Perhaps the biggest statistical challenge that faces microarray studies is the propensity for a high FDR previously defined as false discovery rate caused by multiple hypothesis testing. Current FDR adjustment strategies are dependent on the magnitude of differentially expressed genes, gene expression variability, and sample size [34], and they are often too stringent, resulting in a large number of false negatives. For instance, the application of various FDR adjustment procedures to the interferon stimulated gene set resulted in the loss of all true positives (Table 4). Importantly, the application of LPv showed a very high degree of functional cohesion (LPv 6.0×10^−32^) in the small gene set. The availability of GCAT will provide an objective measure to evaluate the appropriateness of various FDR adjustment methods. Ultimately, it would be very useful to incorporate LSI-derived literature similarities, perhaps as priors in a Bayesian model, to adjust the FDR for microarray datasets. Taken together, GCAT provides a unique tool for genomic researchers to objectively determine the most appropriate analytical approaches for their particular experimental system.

## Supporting Information

Figure S1 **Venn Diagram of differentially expressed genes (DEGs) generated by three different statistical tests on four different microarray datasets: ALL1 (A), ALL2 (B), ALL3 (C) and ALL4 (D).**
(TIF)
