# Supplementary figures and images for: Functional Cohesion of Gene Sets Determined by Latent Semantic Indexing of PubMed Abstracts
Source: PLoS One. 2011 Apr 14;6(4):e18851. doi: 10.1371/journal.pone.0018851 (PMC3077411; doi:10.1371/journal.pone.0018851)

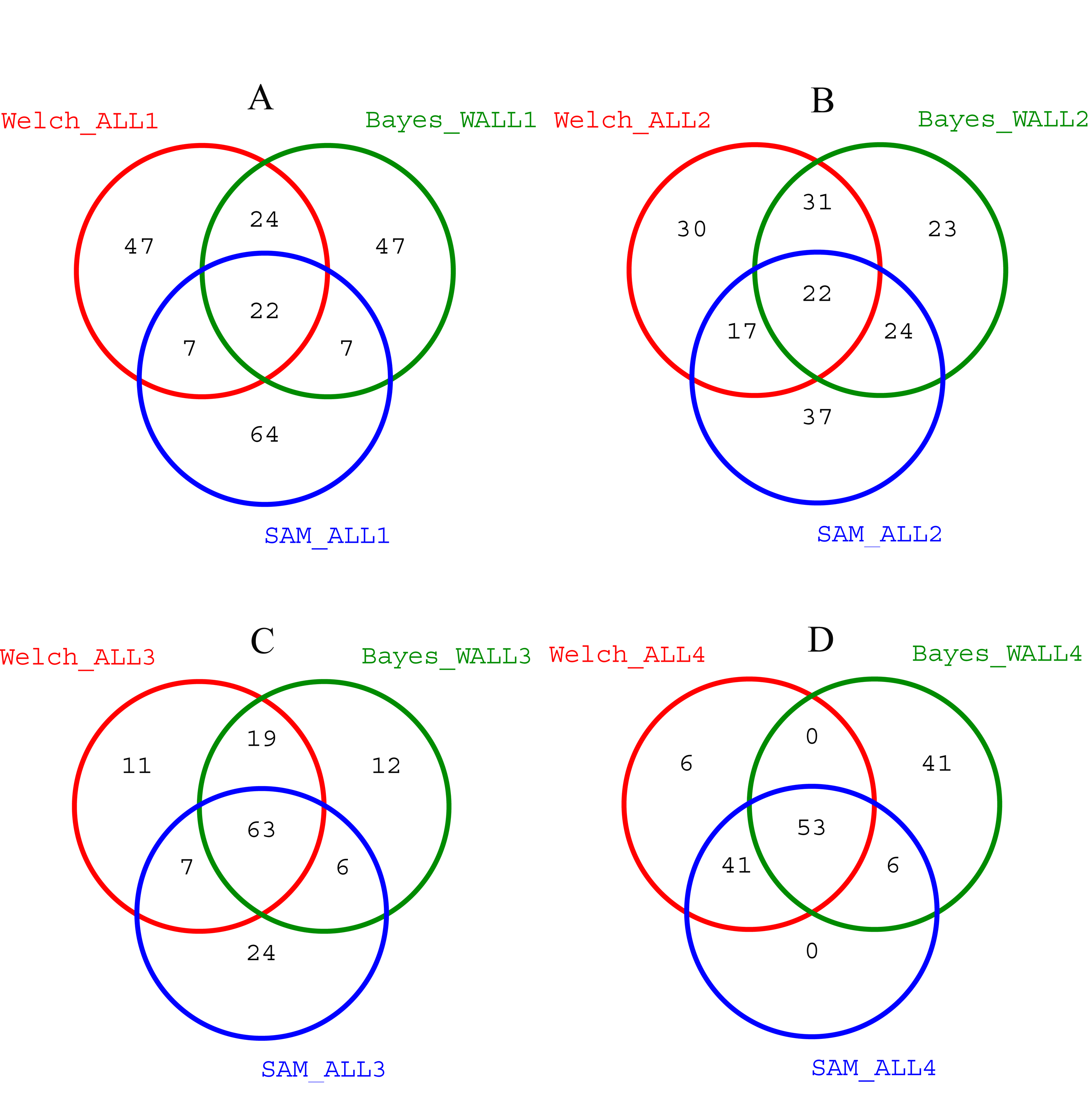

Supplement: Figure S1 — Venn Diagram of differentially expressed genes (DEGs) generated by three different statistical tests on four different microarray datasets: ALL1 (A), ALL2 (B), ALL3 (C) and ALL4 (D). (TIF) [file pone.0018851.s001.tif]
